# *NBN*, *RAD51* and *XRCC3* Polymorphisms as Potential Predictive Biomarkers of Adjuvant Radiotherapy Toxicity in Early HER2-Positive Breast Cancer

**DOI:** 10.3390/cancers14184365

**Published:** 2022-09-08

**Authors:** Katja Goričar, Franja Dugar, Vita Dolžan, Tanja Marinko

**Affiliations:** 1 Pharmacogenetics Laboratory, Institute of Biochemistry and Molecular Genetics, Faculty of Medicine, University of Ljubljana, 1000 Ljubljana, Slovenia; 2 Faculty of Medicine, University of Ljubljana, 1000 Ljubljana, Slovenia; 3 Institute of Oncology Ljubljana, 1000 Ljubljana, Slovenia

**Keywords:** breast cancer, radiotherapy, DNA repair, single nucleotide polymorphism, RAD51, XRCC3

## Abstract

**Simple Summary:**

Adjuvant radiotherapy for breast cancer patients significantly improves survival and causes side effects. It is known that the response to radiotherapy is individual, but we are not yet able to predict patients with high risk for acute or late radiotherapy adverse events. This study aimed to investigate the association between homologous recombination repair (HRR) polymorphisms and radiotherapy toxicity and thus contribute to the knowledge on potential predictive biomarkers of radiotherapy toxicity in early HER2-positive breast cancer. This study was among the first to evaluate the role of HRR genetic variability with cardiac toxicity. *RAD51* polymorphisms were associated with cardiac adverse events, while *XRCC3* polymorphisms were associated with skin adverse events. Our results suggest that polymorphisms in key HRR genes might be used as potential biomarkers of late treatment-related adverse events in early HER2-positive breast cancer treated with radiotherapy.

**Abstract:**

Radiotherapy (RT) for breast cancer significantly impacts patient survival and causes adverse events. Double-strand breaks are the most harmful type of DNA damage associated with RT, which is repaired through homologous recombination (HRR). As genetic variability of DNA repair genes could affect response to RT, we aimed to evaluate the association of polymorphisms in HRR genes with tumor characteristics and the occurrence of RT adverse events in early HER2-positive breast cancer. Our study included 101 breast cancer patients treated with adjuvant RT and trastuzumab. All patients were genotyped for eight single nucleotide polymorphisms in *NBN*, *RAD51* and *XRCC3* using competitive allele-specific PCR. Carriers of *XRCC3* rs1799794 GG genotype were less likely to have higher tumor differentiation grade (OR = 0.05, 95% CI = 0.01–0.44, *p* = 0.007). Carriers of *RAD51* rs1801321 TT genotype were more likely to have higher NYHA class in univariable (OR = 10.0; 95% CI = 1.63–61.33; *p* = 0.013) and multivariable (OR = 9.27; 95% CI = 1.28–67.02; *p* = 0.027) analysis. Carriers of *RAD51* rs12593359 GG genotype were less likely to have higher NYHA class in univariable (OR = 0.09; 95% CI = 0.01–0.79; *p* = 0.030) and multivariable (OR = 0.07; 95% CI = 0.01–0.81; *p* = 0.034) analysis. Carriers of *XRCC3* rs1799794 GG genotypes experienced more skin adverse events based on LENT-SOMA scale in univariable (OR = 5.83; 95% CI = 1.22–28.00; *p* = 0.028) and multivariable (OR = 10.90; 95% CI = 1.61–73.72; *p* = 0.014) analysis. In conclusion, *XRCC3* and *RAD51* polymorphisms might contribute to RT adverse events in early HER2-positive breast cancer patients.

## 1. Introduction

Breast cancer is the most common cancer in women [1]. It is treated with three main types of oncological treatment, including radiotherapy (RT) [1]. RT is a highly successful local treatment that patients receive mostly after surgery on a tumor in the breast. It significantly reduces the chance of recurrence and death from breast cancer [1]. However, like any treatment, it can have side effects [2]. After irradiation of the breast or chest wall, different changes may occur on the irradiated skin and subcutaneous tissue, which affect the aesthetic effect but also cause various difficulties such as pain, fibrosis and swelling of the arm. Moreover, after irradiation of the left breast or thoracic wall, which is located just above the heart, heart diseases such as pericarditis, ischaemic heart disease, arrhythmias or valvular diseases may occur [3]. The vast majority of potential late side effects of RT may deteriorate with ongoing years and thereby significantly impact on patient’s quality of life [2,3]. As RT is often combined with systemic oncological therapy that can also have side effects, a summed toxicity can be even more expressed, as is often the case for skin-related toxicity [2]. On the other hand, as it is with cardiotoxicity, radiation and drugs can have different mechanisms that lead to cardiac side effects. Still, the resulting cardiotoxicity can seriously impact the patient’s quality of life or may even shorten the patient’s life [4].

One of the molecular subtypes of breast cancer is human epidermal growth factor receptor-2 (HER2) positive breast cancer. HER2-positive patients are treated with both systemic therapy and RT [5]. In 2005, a humanized monoclonal antibody targeting the HER2 receptor trastuzumab was added to the adjuvant systemic treatment scheme with tremendous success in prolongation of survival for this subgroup of breast cancer patients [6]. There is a lot of cardiotoxicity research on this subgroup of patients, as both treatment modalities combined with the treatment of HER2-positive breast cancer are potentially cardiotoxic. Additionally, HER2-targeted systemic therapy might exacerbate RT skin side effects [7].

We cannot yet predict which patient will experience more pronounced complications from RT. In clinical practice, patients respond to the same dose of radiation with different grades of skin reactions, even if they do not receive any systemic therapy during their course of oncological treatment. The response to RT is, therefore, highly individual. Thanks to modern oncological treatment, there are many breast cancer survivors. Understanding the mechanisms of occurrence of possible treatment side effects and finding new ways to prevent adverse effects are, therefore, increasingly important [2].

RT exerts its therapeutic effects mainly through the induction of DNA damage [8]. DNA damage leads to cell cycle arrest resulting in either DNA repair, cell death or cell cycle progression [9]. Cancer cells divide more rapidly than normal cells and often have deregulated DNA repair pathways; therefore, they have less time to repair the DNA damage and are more sensitive to the effects of radiation [8]. On the other hand, cancer cells with efficient DNA repair can be resistant to RT [8].

RT induces different types of DNA damage that are repaired through different DNA repair pathways. Most common DNA lesions are modifications of DNA bases repaired by the base excision repair pathway. Additionally, RT induces single-strand breaks and double-strand breaks (DSBs), disrupting the phosphodiester backbone [8,10]. Although less common, DSBs are the most genotoxic and can be produced directly due to ionizing radiation or can occur during replication if initial damage, mainly single-strand breaks, is not repaired [8,10]. Two major pathways are involved in DSB repair, non-homologous end-joining (NHEJ) and homologous recombination repair (HRR) [10]. Several factors can influence which pathway is used [8]. NHEJ is a fast and cell cycle-independent process that does not require a template but is error-prone. On the other hand, HRR is a slower process that can only occur during the cell cycle’s S or G2 phase and requires a sister chromatid as a template. However, a high-fidelity process results in accurately repaired DSB [9,10].

HRR is the best option for maintaining genomic stability, but it requires the concerted action of numerous enzymes. In brief, HRR starts with DSB processing by the MRN complex that consists of nuclease MRE11, RAD50 and nibrin (NBN). MRN initiates 5′ to 3′ DNA end resection that generates 3′ single-strand DNA ends, which are protected from degradation by replication protein A (RPA) [10,11]. MRN and RPA then contribute to the activation of several kinases such as ATM, ATR, CHEK1 and CHEK2 [11]. These kinases enable the activation and recruitment of BRCA1, PALB2 and BRCA2 that exchange RPA with recombinase RAD51, a key protein of HRR that forms a nucleoprotein filament [12]. Several RAD51 paralogs, including XRCC3, facilitate this process [13]. Then, the RAD51 nucleoprotein filament searches for a homologous template in the sister chromatid, leading to strand invasion and elongation. This results in the formation of Holliday junction intermediates, which can be resolved differently, resulting in completely repaired DSBs [13].

Several factors can influence HRR efficiency, including genetic variability. This can affect both cancer susceptibility and treatment response. Notably, hereditary breast cancer is often associated with mutations in tumor suppressor *BRCA1* and *BRCA2* genes [11,14]. However, mutations in other HRR genes, such as *ATM*, *CHEK2*, *PALB2*, *NBN*, *MRE11*, and RAD51 paralogs *RAD51C* and *RAD51D* also increase cancer risk and are already included in many screening panels for breast cancer [11,14,15]. Apart from rare mutations, several common single nucleotide polymorphisms (SNPs) in various HRR genes, including *NBN*, *RAD51*, and *XRCC3*, were reported to affect DNA repair capacity and were previously associated with altered breast cancer risk [16,17,18,19,20,21]. Genetic variability can also influence breast cancer treatment outcomes. For example, carriers of *BRCA1*, *BRCA2* or *PALB2* mutations can benefit from treatment with poly (ADP-ribose) polymerase (PARP) inhibitors [11,22]. Patients with *BRCA1* and *BRCA2* mutations can also be successfully treated with platinum-based chemotherapy [23]. Common SNPs in HRR genes may also influence interindividual differences in RT treatment outcomes and adverse events in different cancer types, but the results can differ among studies [24,25,26,27,28,29].

Our study’s primary aim was to evaluate the association of common putatively functional SNPs in HRR genes *NBN*, *RAD51*, and *XRCC3* with RT adverse events in patients with early HER2-positive breast cancer treated with adjuvant RT. As a secondary aim, we evaluated the association of *NBN*, *RAD51* and *XRCC3* SNPs with tumor differentiation grade and occurrence of a new primary tumor after longer follow-up in patients with early HER2-positive breast cancer.

## 2. Materials and Methods

### 2.1. Patients

Our retrospective genetic association study with a longitudinal follow-up included patients with early HER2-positive left- or right-sided breast cancer (stage I–III). They were treated concurrently with trastuzumab and RT at the Institute of Oncology Ljubljana between June 2005 and December 2010. HER2 status of the tumor and the primary tumor differentiation grade according to the Nottingham histological grading were determined according to our standard clinical practice [30]. Patients received adjuvant treatment according to clinical guidelines, namely surgery, chemotherapy, endocrine therapy in case of hormone receptor-positive disease, trastuzumab and RT. Trastuzumab treatment started before RT or on the first day of RT at the latest. After completing adjuvant treatment, an outpatient follow-up visit was scheduled, during which patients completed a survey on smoking, comorbidities and cardiovascular disease. At the examination, the adverse events of the treatment on the irradiated region and any potential heart-related problems were assessed. All patients also had follow-up echocardiography to reveal potential cardiac adverse events, and laboratory cardiac parameters were measured in the blood. In 2021, we analyzed the vital status of the patients, any locoregional or distant recurrence of primary breast cancer or the occurrence of any other new primary tumor.

The study was registered at ClinicalTrial.gov (identifier NCT 01572883) and conducted in accordance with the Declaration of Helsinki. All participants signed informed consent before participating in the study approved by the Republic of Slovenia National Medical Ethics Committee (approval number 39/05/15, 0120-54/2015-2, 0120-54/2015-11).

#### 2.1.1. Systemic Treatment

Data on systemic oncology treatment were obtained from patient records. Patients with an indication for systemic chemotherapy were mainly treated with anthracyclines and taxanes, which were prescribed in the following regimens: Option 1: 4 cycles of epirubicin plus cyclophosphamide (EC) or doxorubicin plus cyclophosphamide (AC) every 3 weeks, followed by 12 cycles of paclitaxel weekly; Option 2: 4 cycles of EC or AC every 3 weeks, followed by 3 cycles of docetaxel every 3 weeks; or Option 3: 3 to 4 cycles of 5-fluorouracil, epirubicin and cyclophosphamide (FEC) or doxorubicin in combination with 5-fluorouracil and cyclophosphamide (FAC) every 3 weeks, followed by 3–4 cycles of docetaxel every 3 weeks. The indications for trastuzumab treatment were set according to the pivotal clinical trials. In the case of negative axillary lymph nodes, patients received trastuzumab only if the tumor was larger than 2 cm, while in the case of positive axillary lymph nodes, patients received trastuzumab in any case [6,30]. The WHO performance status of zero or one, no serious concomitant cardiac disease, and treatment with adjuvant chemotherapy was also a prerequisite for adjuvant trastuzumab therapy [6,30]. Treatment with trastuzumab started 3 weeks after the last cycle of anthracyclines and was prescribed for 1 year.

#### 2.1.2. Locoregional Treatment

Locoregional treatment was carried out according to clinical guidelines. For the majority of patients, either breast-conserving surgery or mastectomy was performed as the first step of treatment, with concomitant removal of the sentinel lymph node or axillary dissection in the ipsilateral axilla. After adjuvant chemotherapy, patients received RT. In all cases, breast-conserving surgery was an indication for the irradiation of the operated breast, while after mastectomy, patients were irradiated to the chest wall only if the tumor was ≥5 cm. In addition to irradiating the breast/chest wall, all patients with 4 or more positive axillary lymph nodes also received regional RT to the periclavicular and supraclavicular lymph nodes. RT parameters are specified in Section 3.1.

### 2.2. Assessment of Adverse Events

#### 2.2.1. Cardiac Adverse Events

Cardiac adverse events were assessed using New York Heart Association (NYHA) classification to assess signs of heart failure [31], echocardiography with left ventricular ejection fraction (LVEF) measurement, and measurement of serum N-terminal pro-B-type natriuretic peptide (NT-proBNP) concentration. Echocardiography with LVEF measurement was performed before treatment with adjuvant RT and after the completed treatment with adjuvant RT and trastuzumab. Baseline LVEF was determined with echocardiography or radionuclide ventriculography as previously described, with LVEF values of 50% or more considered normal [30]. Absolute change in LVEF was calculated as the difference between LVEF after treatment and LVEF before RT. Important LVEF reduction was classified as a decrease of LVEF for 10 percentage points or more or as a final value of LVEF below 50% [4]. Serum NT-proBNP was measured using the Cobas e 411 analyzer (Roche, Switzerland) according to the standard clinical practice at the follow-up clinical examination treatment with adjuvant RT and trastuzumab [30]. The values of NT-proBNP below 125 ng/L were considered normal (no heart failure) based on the recommendations of the European Society of Cardiology for the non-acute setting [32].

#### 2.2.2. Skin Adverse Events

Late skin adverse events were evaluated using Common Terminology Criteria for Adverse Events, version 3.0 (CTCAE v.3) evaluating skin hyperpigmentation, atrophy, induration and telangiectasia [33] and Late Effects in Normal Tissues/Subjective, Objective, Management and Analytic (LENT-SOMA) criteria evaluating skin atrophy, fibrosis, and ulceration as well as pain, edema and telangiectasia [34]. Skin adverse events were defined as adverse events grade 2 or higher.

### 2.3. DNA Extraction, Tag SNP Selection and Genotyping

Genomic DNA was extracted from buccal swab samples (INFINITI Buccal Sample Collection Kit, AutoGenomics Inc., Vista, CA, USA) using QIAamp DNA Mini Kit (QIAGEN, Hilden, Germany) following the protocol provided by the manufacturer.

Our study focused on the genetic variability of key HRR genes previously reported in the literature [16,17,18,19,20,21]. Putatively functional tag single nucleotide polymorphisms (SNPs) in genes *XRCC3*, *RAD51* and *NBN* were selected based on the data from the International HapMap Project [35]. Only SNPs in the coding region, 5′ or 3′ untranslated regions with minor allele frequency above 5% in the European population were included in the study. One SNP was chosen for the analysis from each haplotype block with high linkage disequilibrium (R^2^ > 0.8). All patients were genotyped for eight tag SNPs using fluorescent-based competitive allele-specific polymerase chain reaction (KASP assay, LGC Genomics, UK) according to the manufacturer’s instructions.

### 2.4. Statistical Analysis

Median and interquartile ranges (25–75%) were used to describe continuous variables, while frequencies were used to describe categorical variables. For all SNPs, the chi-square test evaluated deviation from Hardy–Weinberg equilibrium (HWE). Additive and dominant genetic models were used in the analyses. To evaluate the association of selected SNPs with tumor differentiation, markers of cardiotoxicity, skin toxicity, and occurrence of a new primary tumor, univariable and multivariable logistic regression was used to calculate odds ratios (ORs) and corresponding 95% confidence intervals (CIs). Clinical parameters used for adjustment in multivariable analysis were selected using stepwise forward-conditional logistic regression. If there were no patients in one of the groups, Fisher’s exact test was used to compare genotype frequencies. Statistical analyses were performed with IBM SPSS Statistics version 27.0 (IBM Corporation, Armonk, NY, USA). Haplotypes were reconstructed to evaluate the combined effect of more SNPs within one gene using Thesias version 3.1, where the most common haplotype was used as a reference [36]. All statistical tests were two-sided. As eight SNPs from three genes were investigated in our study, Bonferroni correction was used to account for multiple comparisons: *p*-values below 0.006 were considered statistically significant, while *p*-values between 0.006 and 0.050 were considered nominally significant. For SNPs with a minor allele frequency of 30–40%, this study had 80% power to detect ORs of 3.3 or more for more frequent adverse events and ORs above 4.5 or 5.8 for less frequent adverse events. Power calculation was performed by the PS Power and sample size calculations, version 3.1.2 [37].

## 3. Results

### 3.1. Patients’ Characteristics

The study included 101 patients with early HER2-positive breast cancer. Overall, 96 patients (95.0%) had invasive ductal carcinoma, and 69 (68.3%) cases were histological grade 3. Their clinical characteristics are presented in Table 1.

All patients received adjuvant RT and trastuzumab, while 99 (98.0%) patients also received anthracyclines and 58 (57.4%) received taxanes. Hormonal therapy was given to 57 (56.4%) patients. All treatment was administered according to the established clinical guidelines. Most of the patients (80, 79.2%) were treated with two-dimensional (2D) RT, and 84 (83.2%) were irradiated with a 25 × 2 Gy scheme. In addition to irradiation of the mammary region, regional lymph nodes were irradiated in 43 (42.6%) patients. Detailed treatment parameters are presented in Table 2.

Data on adverse events and treatment outcomes are presented in Table 3. In all patients, late skin and cardiac adverse events were evaluated after treatment, at the median follow-up after the beginning of RT of 4.0 (2.6–5.4) years. Regarding markers of cardiotoxicity, 36 (35.6%) patients had increased serum NT-proBNP, with a median level of 90 (56–157) ng/L. Additionally, 17 (16.8%) patients had mild symptoms of heart failure (NYHA class 2), while clinically important LVEF reduction was observed only in 9 (8.9%) patients. We observed skin adverse events grade 2 or more according to LENT-SOMA criteria in 33 (32.7%) patients, while skin toxicity grade 2 or more according to CTCAE v.3 was observed in 12 (11.9%) patients. Data regarding tumor recurrence, the occurrence of a new primary tumor and vital status were assessed at the median follow-up of 13.5 (11.9–15.1) years after RT. Altogether there were 3 (3.0%) distant recurrences and 0 loco-regional recurrences. Additionally, 9 (8.9%) patients were diagnosed with a new tumor, while 2 (2.0%) patients died.

Eight tag polymorphisms were selected for genotyping: *NBN* rs1805794 (p.Glu185Gln), *NBN* rs709816 (p.Asp399=), *NBN* rs1063054 (c.*1209A>C), *RAD51* rs1801320 (c.-98G>C), *RAD51* rs1801321 (c.-61G>T), *RAD51* rs12593359 (c.*502T>G), *XRCC3* rs1799794 (c.-316A>G), and *XRCC3* rs861539 (p.Thr241Met). Genotype and minor allele frequencies of selected SNPs are shown in Table 4. The genotype frequencies of all SNPs were consistent with HWE. Experimentally confirmed or putative *in silico* predicted functional effect of selected polymorphisms is presented in Table 4.

### 3.2. Association of Selected SNPs with Tumor Differentiation Grade

Carriers of *XRCC3* rs1799794 GG genotype were less likely to have grade 3 tumor compared to carriers of wild-type AA genotype (OR = 0.05, 95% CI = 0.01–0.44, *p* = 0.007). No significant association was found between other SNPs and histological grade (Table 5). Smoking, age or BMI were not associated with tumor differentiation grade (all *p* > 0.05).

### 3.3. Association of Selected SNPs with Cardiac Adverse Events

Among clinical parameters, chemotherapy scheme, hormonal therapy, other treatment parameters or smoking were not statistically significantly associated with observed differences in NYHA class in our study group (all *p* > 0.05). Higher age was associated with higher NYHA class (OR = 1.06, 95% CI = 1.00–1.12, *p* = 0.048), but only in univariable analysis. In a multivariable model, a significant association with higher NYHA class for both hyperlipidemia (OR = 4.60, 95% CI = 1.39–15.19, *p* = 0.012) and body mass index (BMI) (OR = 1.20, 95% CI = 1.05–1.38, *p* = 0.006).

Carriers of *RAD51* rs1801321 TT genotype were more likely higher NYHA class in the univariable analysis (OR = 10.0, 95% CI = 1.63–61.33, *p* = 0.013) and after adjustment for hyperlipidemia and BMI (OR = 9.27, 95% CI = 1.28–67.02, *p* = 0.027). However, the risk for higher NYHA class was nominally significantly decreased in carriers of *RAD51* rs12593359 GG genotype in the univariable (OR = 0.09, 95% CI = 0.01–0.79, *p* = 0.030) and multivariable (OR = 0.07, 95% CI = 0.01–0.81, *p* = 0.034) analysis (Table 6).

Detailed results regarding the association with NT-proBNP and LVEF reduction are shown in Table S1. Higher age was significantly associated with serum level of NT-proBNP above 125 ng/L (OR = 1.05, 95% CI = 1.01–1.09, *p* = 0.023). No other clinical parameter, including treatment parameters, smoking or BMI, was statistically significantly associated with our study group’s observed proportion of patients with increased NT-proBNP (all *p* > 0.05). However, no significant association with increased NT-proBNP was found for selected polymorphisms in univariable or multivariable analysis. No clinical parameter, including treatment parameters, smoking, age or BMI, was statistically significantly associated with the observed proportion of patients with LVEF reduction in our study group (all *p* > 0.05). Carriers of *NBN* rs1063054 AC genotype were more likely to experience LVEF reduction, but the association was not significant or nominally significant (OR = 7.18, 95% CI = 0.84–60.99, *p* = 0.071) (Table S1).

We also performed a haplotype analysis to assess the combined effect of all selected *RAD51* SNPs on NYHA class (Table S2). The observed haplotypes in our study were *RAD51* GGG, GTT and CGT, with their estimated frequencies of 0.453, 0.360 and 0.116, respectively. Carriers of the *RAD51* GTT haplotype were significantly more likely to present higher NYHA class than the reference *RAD51* GGG haplotype (OR = 4.27, 95% CI = 1.45–12.58, *p* = 0.009). The association remained nominally significant even after adjustment for BMI (OR = 3.69, 95% CI = 1.24–11.02, *p* = 0.019) or hyperlipidemia (OR = 4.37, 95% CI = 1.33–14.35, *p* = 0.015).

### 3.4. Association of Selected SNPs with Skin Adverse Events

Among clinical parameters, arterial hypertension was significantly associated with higher grade of skin adverse events according to both LENT-SOMA scale (OR = 8.44, 95% CI = 2.57–27.79, *p* < 0.001) and CTCAE v.3 scale (OR = 5.38, 95% CI = 1.36–21.26, *p* = 0.016). Similarly, treatment with taxanes was significantly associated with higher grade of skin adverse effects according to LENT-SOMA (OR = 7.14, 95% CI = 2.14–23.87, *p* = 0.001) and CTCAE v.3 (OR = 16.23, 95% CI = 1.83–144.0, *p* = 0.012). Other clinical parameters, including treatment parameters, smoking or BMI, were not statistically significantly associated with the observed severity of skin adverse events according to either scale in our study group (all *p* > 0.05).

Regarding the LENT-SOMA scale, the risk for late adverse events was nominally significantly increased in carriers of *XRCC3* rs1799794 GG genotype in univariable (OR = 5.83, 95% CI = 1.22–28.00, *p* = 0.028) and multivariable (OR = 10.90, 95% CI = 1.61–73.72, *p* = 0.014) analysis. On the other hand, carriers of *XRCC3* rs861539 CT and TT genotypes tended to have a decreased risk of skin adverse events only in univariable analysis (OR = 0.43, 95% CI = 0.18–1.00, *p* = 0.050). No association was found between selected SNPs and adverse events according to CTCAE criteria (Table 7).

In haplotype analysis, we evaluated the combined effect of both *XRCC3* SNPs on the occurrence of skin adverse events according to the LENT-SOMA criteria (Table S3). The estimated frequencies of the *XRCC3* AC, AT and GC haplotypes were 0.386, 0.342 and 0.272, respectively. Carriers of *XRCC3* GC haplotype were slightly more likely to develop skin adverse events, but the association was not significant (OR = 1.93, 95% CI = 0.94–3.98, *p* = 0.074), not even after adjustment for clinical parameters (arterial hypertension, treatment with taxanes) (OR = 2.06, 95% CI = 0.87–4.85, *p* = 0.100).

### 3.5. Association of Selected SNPs with the Occurrence of a New Primary Tumor

New primary tumor occurred in 9 (8.9%) of patients: two patients had a new breast tumor, while colon adenocarcinoma, lung carcinoma, pituitary carcinoma, bladder carcinoma, cutaneous squamous cell carcinoma, uterine carcinosarcoma and endometrial carcinoma occurred in one patient each. No clinical or treatment parameter, including smoking, age or BMI, was significantly associated with the occurrence of a new tumor (all *p* > 0.05). Among investigated SNPs, only *RAD51* rs12593359 tended to be associated with the occurrence of a new primary tumor (Table 8): 4 (18.2%) carriers of GG genotype developed a new primary tumor, compared to no carriers of wild-type AA genotype (*p* = 0.049).

## 4. Discussion

In the present study, we evaluated the association of tag SNPs in HRR genes *NBN*, *RAD51* and *XRCC3* with toxicity and outcome of adjuvant RT and tumor differentiation grade in early HER2-positive breast cancer patients. *RAD51* polymorphisms were associated with symptoms of heart failure according to NYHA class and the occurrence of a new primary tumor, while *XRCC3* polymorphisms were associated with tumor differentiation and skin adverse events according to LENT-SOMA criteria.

Adverse events can influence the outcome of RT treatment. Among the main potential radiation-induced adverse events in breast cancer patients are skin toxicity, cardiotoxicity and pulmonary toxicity [44]. Our study evaluated the association of investigated SNPs with late treatment-related skin and cardiac adverse events. Cardiac adverse events have been observed after both RT and systemic treatment. They have become a focus of recent research due to their important influence on the therapeutic benefits of modern clinical practice [45], as early HER2-positive breast cancer is always treated with a combination of chemotherapy and HER2 targeted therapy as well as hormonal therapy in hormonal positive cases. Studying the side effects of adjuvant radiotherapy in this cohort of patients that are never treated with RT alone is challenging.

Regarding cardiac adverse events in our study, the most common marker was increased serum NT-proBNP. Some patients also exhibited mild heart failure symptoms, while clinically important LVEF reduction was rare. Among the investigated SNPs, only *RAD51* genetic variability was associated with NYHA class, even after adjusting for the presence of hyperlipidemia and higher BMI that were associated with higher NYHA class. On the other hand, no associations were observed with NT-proBNP or LVEF. Regarding skin adverse events, more patients reported adverse events according to LENT-SOMA criteria than CTCAE v.3 criteria in our study. Among the investigated SNPs, only *XRCC3* genetic variability was associated with LENT-SOMA grade in a single SNP analysis. Some associations remained nominally significant after adjustment for arterial hypertension and treatment with taxanes, both associated with a higher grade of skin adverse events. On the other hand, no associations were observed with CTCAE. *NBN* genetic variability was also not associated with any of the investigated adverse events.

In our study, carriers of *RAD51* rs1801321 TT genotype more often had higher NYHA class, while carriers of *RAD51* rs12593359 GG genotype were less likely to exhibit symptoms of heart failure in both single SNP and haplotype analysis. *RAD51* rs1801321 (c.-61G>T) is located in the 5′ untranslated regions of the promoter, and it might affect transcription factor binding and, consequently, RAD51 expression. Previously, enhanced promoter activity was reported for the polymorphic rs1801321 T allele [41]. In previous studies investigating the association of *RAD51* rs1801321 with response to RT, no association was observed with radiation pneumonitis in lung cancer [46], skin toxicity or mucositis in head and neck cancer [47] or fibrosis in oropharyngeal carcinoma [48]. This is consistent with our results regarding skin adverse events. On the other hand, cardiac toxicity’s role was not yet investigated.

*RAD51* rs12593359 (c.*502T>G) is located in the 3′ untranslated region, and it was proposed that it affects the miRNA binding site for miR-129-3p and thus influences post-transcriptional regulation of RAD51 [42]. In cell lines, the GG genotype was associated with decreased *RAD51* mRNA expression [42]. Thus far, only a handful of studies have investigated the role of this SNP, where it was not associated with cancer risk [49,50,51] or response to platinum-based chemotherapy [52,53]. On the other hand, no studies have investigated the response to RT or cardiac-related phenotypes yet.

*RAD51* rs1801320 (c.-98G>C) is another frequently studied promoter polymorphism associated with enhanced promoter activity in one study [41]. At the same time, another study observed a different effect for different isoforms, where the polymorphic C allele was associated with splicing and decreased mRNA expression of isoform 2 transcripts [54], suggesting its role might be more complex. In our study, it was not an important predictor of RT outcome, which is consistent with the results of previous studies on breast, head and neck cancer [28,47,55]. On the other hand, *RAD51* rs1801320 was associated with radiation pneumonitis in lung cancer [46] and radiochemotherapy-induced acute toxicity in rectal cancer [24].

The results of different studies therefore suggest that the role of RAD51 in RT treatment outcome is complex. Increased expression of RAD51 can be associated with lower radiosensitivity due to more efficient DNA repair. However, it could also lead to uncontrolled HRR and genomic instability [15], influencing the effects on normal tissues. Several transcription factors affect RAD51 expression, and *BRCA1* and *BRCA2* mutations could also modify the expression and the role of RAD51 [15]. RAD51 was also proposed as a potential therapeutic target in cancer [15]. Interestingly, deficiency of BRCA2 was previously associated with decreased RAD51 focus formation, decreased repair of DNA damage, and cardiotoxicity of doxorubicin in animal models [56]. Still, the role of RAD51 in cardiac adverse events is largely unexplored. Therefore, further studies are needed to evaluate the functional role of *RAD51* SNPs and their association with cardiac adverse events of RT.

In our study, carriers of *XRCC3* rs1799794 GG genotypes had an increased risk for late skin adverse events after RT, while carriers of *XRCC3* rs861539 CT and TT genotypes tended to have fewer skin adverse events. However, the combination of both polymorphisms in haplotype analysis was not significant. *XRCC3* rs1799794 (c.-316A>G) is located in the 5′ untranslated region, and it might affect transcription factor binding, but the functional role is not well established. The results regarding the role of this SNP and RT adverse events vary among studies [28,57,58,59,60]. However, in a meta-analysis combining different cancer types, this SNP was associated with decreased risk for late RT toxicity, while no association with acute toxicity was observed [26]. These results differ from ours; however, they are based on only three studies on head and neck cancer, gynecological, and breast cancer [26]. In the study on breast cancer, the association was not statistically significant [28]. These differences could be due to different treatment regimens, differences among populations or different times of follow-up; therefore, further studies are needed in this area.

*XRCC3* rs861539 (p.Thr241Met) is a non-synonymous SNP in the ATP-binding domain of the protein that affects XRCC3 interactions with other proteins [43]. Several studies investigated the role of this SNP in response to RT in different cancer types. Some studies reported that the polymorphic T allele confers an increased risk for fibrosis or telangiectasia [25,48,61], erythema and acute skin toxicity [55,62] in breast and other cancers. Still, other studies did not replicate the results, especially for late toxicity [27,28,63,64,65]. Similarly, in a meta-analysis, a significant association of *XRCC3* rs861539 with increased acute RT toxicity was observed, while the association with late adverse events did not reach statistical significance. In our study, a trend was observed only in univariable analysis, suggesting that this SNP does not contribute importantly to late skin adverse events of RT.

Proteins involved in HRR can also affect tumor characteristics. DNA damage accumulates, leading to genomic instability and can affect cell differentiation [66]. For example, high *RAD51* gene expression was associated with aggressiveness, metastasis, and poor survival in breast cancer [67]. The highest *RAD51* expression was observed in triple-negative breast cancer, the most aggressive breast cancer subtype, compared to all other immunohistochemical breast cancer subtypes [67]. Overexpression of RAD51 was also associated with the histological classification of invasive ductal carcinoma of the breast and was proposed as a potential diagnostic and prognostic biomarker [68]. We, therefore, investigated the association of HRR SNPs with tumor differentiation grade. In our study, carriers of *XRCC3* rs1799794 GG genotype were less likely to have grade 3 tumors than carriers of AA genotype. No significant association was found between other SNPs and histological grade. Several studies evaluated the association of DNA repair polymorphisms in the literature with the histopathological characteristics of breast tumors [66,69,70,71,72]. In one previous study, *XRCC3* rs1799794 was not associated with tumor grade [66], contrary to our results; however, the study included both HER2-positive and negative patients. *XRCC3* rs861539 was associated with tumor grade in one study, with different results for carriers of one or two T alleles [70]. Still, other studies investigating HRR polymorphisms also did not observe any significant associations with tumor grade, similar to our results [66,69,71,72].

Our cohort of patients also had a high incidence (9.8%) of new primary tumors that unexpectedly exceeded the incidence (3.0%) of primary breast cancer recurrences. Among the studied SNPs, only *RAD51* rs12593359 was associated with the occurrence of a new primary tumor: 18.2% of carriers of the GG genotype got a new primary tumor. In contrast, carriers of AA genotype did not. This polymorphism was previously not associated with cancer risk [49,50,51]. Even though data regarding the influence of genetic variability on the occurrence of a new tumor are scarce, there are many reports in the literature that DNA repair genes, including *XRCC3* and *RAD51*, are associated with the development of breast cancer and other cancer types. One of the most investigated SNP is *XRCC3* rs861539, and several studies and meta-analyses suggest it may contribute to breast cancer risk. Still, the effect can differ among populations and cancer types [16,17,73,74]. *XRCC3* rs1799794 was also associated with increased breast cancer risk [18]. According to the literature, *RAD51* rs1801320 was also associated with increased risk for breast and other cancers, especially in carriers of *BRCA2* mutations. However, differences were observed among populations and cancer types [20,74,75,76]. In our study, these SNPs were not associated with new primary tumor occurrence, but further studies are needed in this field.

Our study also has limitations, such as small sample size and limited observation period. Since most of the patients were irradiated during the period when 2D RT was the standard technique in our institution and three-dimensional (3D) RT was just being introduced, the findings of our study should be verified in a group of patients with early HER2-positive breast cancer treated with modern irradiation techniques such as 3D RT, Intensity-Modulated Radiation Therapy (IMRT) or Volumetric Modulated Arc Therapy (VMAT). Although HER2-positive breast cancer is an aggressive type of cancer, we didn’t observe many relapses of breast cancer in our patients. Due to small numbers of disease recurrence or death in our study during the observation period, we could not evaluate the association of SNPs with these outcomes. On the other hand, our study included a clinically well-defined group of early HER2-positive breast cancer patients who evaluated different adverse events and treatment outcomes after longer follow-ups. Particularly the association of genetic variability with cardiac adverse events of RT in breast cancer was so far largely unexplored. We also used the tag SNP approach to cover most of the genetic variability within a specific gene and used haplotype analysis to evaluate their combined effect. Another limitation of our study is that no data on *BRCA1* or *BRCA2* mutations were available due to ethical reasons. As BRCA1 and BRCA2 play an important role in HRR and breast cancer risk, their interaction with genes included in our study regarding the investigated outcomes would be of great interest for future studies. Additionally, SNPs in genes involved in other DNA repair pathways, such as base excision repair, might contribute to the occurrence of RT adverse events [77]. For example, a combination of several SNPs in polygenic risk scores might better explain the interindividual differences in radiosensitivity [78].

To the best of our knowledge, our study is the first to evaluate the role of HRR polymorphisms in cardiac adverse events in early HER2-positive breast cancer. Further larger prospective studies with modern RT treatment techniques are needed to confirm our observations. Additionally, systemic therapy can contribute to differences in the occurrence of adverse events. Future studies on other breast cancer subtypes treated with RT alone could provide further insight regarding the role of investigated SNPs in HRR.

## 5. Conclusions

In conclusion, our results suggest that selected SNPs in key HRR genes might be potential biomarkers of late treatment-related adverse events in early HER2-positive breast cancer. *RAD51* polymorphisms were mostly associated with cardiac adverse events, while *XRCC3* polymorphisms were associated with skin adverse events. Additionally, selected SNPs in key HRR genes might be associated with breast tumor characteristics. *RAD51* polymorphisms were also associated with the occurrence of a new primary tumor, while *XRCC3* polymorphisms were associated with tumor differentiation grade. In the future, if confirmed in larger studies, genetic factors might help identify patients with higher risk for acute or late RT adverse events, enabling more tailored management and treatment of breast cancer patients. This may potentially improve treatment outcomes and quality of life.

## Figures and Tables

**Table 1:** Clinical characteristics of breast cancer patients included in the study (N = 101).

Characteristic	Category/Unit	N (%)
Age	Years	50.9 (42.1–59.1) ^1^
Body mass index	kg/m^2^	27.1 (24.3–29.7) ^1^
Smoking	Yes	16 (15.8)
	No	85 (84.2)
Diabetes	Yes	1 (1.0)
	No	100 (99.0)
Arterial hypertension	Yes	29 (28.7)
	No	72 (71.3)
Hyperlipidemia	Yes	21 (20.8)
	No	80 (79.2)
Tumor type	Invasive ductal carcinoma	96 (95.0)
	Invasive lobular carcinoma	2 (2.0)
	Other	3 (3.0)
Tumor differentiation grade	1	1 (1.0)
	2	31 (30.7)
	3	69 (68.3)

^1^ median (25–75%).

**Table 2:** Treatment parameters of breast cancer patients included in the study (N = 101).

Characteristic	Category/Unit	N (%)
Type of surgery	Conservative surgery	53 (52.5)
	Mastectomy	48 (47.5)
Side of surgery	Right	53 (52.5)
	Left	48 (47.5)
Chemotherapy scheme	AC/EC/FAC/FEC with taxanes	54 (53.5)
	AC/EC/FAC/FEC without taxanes	43 (42.6)
	Other	4 (4.0)
Taxanes	Docetaxel	41 (40.6)
	Paclitaxel	17 (16.7)
	No	43 (42.6)
Anthracyclines	Epirubicin	93 (92.1)
	Doxorubicin	6 (6.0)
	No	2 (2.0)
Hormonal therapy	Yes	57 (56.4)
	No	44 (43.6)
Site of RT	Breast/mammary region	58 (57.4)
	(Breast/mammary region) + regional lymph nodes	43 (42.6)
RT technique	2D RT	80 (79.2)
	3D CRT	14 (13.9)
	Electrons to the chest wall	7 (6.9)
Treatment scheme of RT	25 × 2 Gy	84 (83.2)
	17 or 18 × 2.5 Gy	17 (16.8)

2D RT, Two-dimensional radiotherapy; 3D CRT, Three-dimensional conformal radiotherapy; AC, doxorubicin, cyclophosphamide; BSA, body surface area calculated according to the Du Bois formula; EC, epirubicin, cyclophosphamide; FAC, 5-fluorouracil, doxorubicin, cyclophosphamide; FEC, 5-fluorouracil, epirubicin, and cyclophosphamide; RT, radiotherapy.

**Table 3:** Markers of late cardiac and skin adverse events of breast cancer therapy and treatment outcome.

	Marker	Category	N (%)
Cardiac adverse events markers	NT-proBNP	<125 ng/L	65 (64.4)
		≥125 ng/L	36 (35.6)
	NYHA	Class 1	84 (83.2)
		Class 2	17 (16.8)
	LVEF reduction	No	92 (91.1)
		Yes	9 (8.9)
Skin adverse events	LENT-SOMA	Grade 1	68 (67.3)
		Grade 2	31 (30.7)
		Grade 3	2 (2.0)
	CTCAE v.3	Grade 1	89 (88.1)
		Grade 2	12 (11.9)
Treatment outcome	Disease recurrence	No	98 (97.0)
		Yes	3 (3.0)
	New primary tumor	No	92 (91.1)
		Yes	9 (8.9)
	Death	No	99 (98.0)
		Yes	2 (2.0)

CTCAE v.3., Common Terminology Criteria for Adverse Events, version 3.0; LENT SOMA, Late Effects in Normal Tissues/Subjective, Objective, Management and Analytic; LVEF, left ventricular ejection fraction, NT-proBNP, N-terminal pro B-type natriuretic peptide; NYHA: New York Heart Association.

**Table 4:** Genotype frequencies of selected polymorphisms and their functional effect.

Gene	SNP	DNA Change ^†^	Protein Change ^†^	Functional Effect	Genotype	N (%)	MAF	pHWE
*NBN*	rs1805794	NM_002485.5: c.553G>C	NP_002476.2: p.Glu185Gln	nsSNP, may influence splicing [38] and may affect interactions with other proteins [39]	CC	47 (46.5)	0.31	0.479
					CG	46 (45.5)		
					GG	8 (7.9)		
*NBN*	rs709816	NM_002485.5: c.1197A>G	NP_002476.2: p.Asp399=	May influence splicing [40]	AA	39 (38.6)	0.36	0.219
					AG	52 (51.5)		
					GG	10 (9.9)		
*NBN*	rs1063054	NM_002485.5: c.*1209A>C	/	May affect miRNA binding [38]	AA	42 (41.6)	0.35	0.835
					AC	47 (46.5)		
					CC	12 (11.9)		
*RAD51*	rs1801320	NM_002875.5: c.-98G>C	/	May affect TF binding, affects promoter activity [41]	GG	73 (72.3)	0.14	0.106
					GC	28 (27.7)		
					CC	0 (0.0)		
*RAD51*	rs1801321	NM_002875.5: c.-61G>T	/	May affect TF binding, affects promoter activity [41]	GG	34 (33.7)	0.40	0.237
					GT	54 (53.5)		
					TT	13 (12.9)		
*RAD51*	rs12593359	NM_002875.5: c.*502T>G	/	May affect miRNA binding [38,42]	TT	23 (22.8)	0.50	0.273
					GT	56 (55.4)		
					GG	22 (21.8)		
*XRCC3*	rs1799794	NM_005432.4: c.-316A>G	/	May affect TF binding [38]	AA	54 (53.5)	0.27	0.797
					AG	39 (38.6)		
					GG	8 (7.9)		
*XRCC3*	rs861539	NM_005432.4: c.722C>T	NP_005423.1: p.Thr241Met	nsSNP, may influence splicing [38] and may affect interactions with other proteins [43]	CC	44 (43.6)	0.34	0.924
					CT	45 (44.6)		
					TT	12 (11.9)		

HWE, Hardy–Weinberg equilibrium; MAF, minor allele frequency; ns, non-synonymous; SNP, single nucleotide polymorphism; TF, transcription factor. **^†^** labeled according to Human Genome Variation Society (HGVS) nomenclature.

**Table 5:** Association of investigated polymorphisms in HRR genes with tumor differentiation grade.

SNP	Genotype	Grade 1 + 2 N (%)	Grade 3 N (%)	OR (95% CI)	*p*
*NBN* rs1805794	CC	16 (34.0)	31 (66.0)	Ref.	
	CG	12 (26.1)	34 (73.9)	1. 46 (0.60–3.57)	0.404
	GG	4 (50.0)	4 (50.0)	0.52 (0.11–2.34)	0.391
	CG + GG	16 (29.6)	38 (70.4)	1.23 (0.53–2.84)	0.635
*NBN* rs709816	AA	13 (33.3)	26 (66.7)	Ref.	
	AG	15 (28.8)	37 (71.2)	1.23 (0.50–3.02)	0.646
	GG	4 (40.0)	6 (60.0)	0.75 (0.18–3.13)	0.693
	AG + GG	19 (30.6)	43 (69.4)	1.13 (0.48–2.67)	0.777
*NBN* rs1063054	AA	17 (40.5)	25 (59.5)	Ref.	
	AC	12 (25.5)	35 (74.5)	1.98 (0.81–4.88)	0.136
	CC	3 (25.0)	9 (75.0)	2.04 (0.48–8.65)	0.333
	AC + CC	15 (25.4)	44 (74.6)	2.00 (0.85–4.67)	0.111
*RAD51* rs1801320	GG	20 (27.4)	53 (72.6)	Ref.	
	GC	12 (42.9)	16 (57.1)	0.50 (0.20–1.25)	0.138
*RAD51* rs1801321	GG	11 (32.4)	23 (67.6)	Ref.	
	GT	14 (25.9)	40 (74.1)	1.37 (0.53–3.50)	0.516
	TT	7 (53.8)	6 (46.2)	0.41 (0.11–1.51)	0.181
	GT + TT	21 (31.3)	46 (68.7)	1.05 (0.43–2.54)	0.918
*RAD51* rs12593359	TT	10 (43.5)	13 (56.5)	Ref.	
	GT	16 (28.6)	40 (71.4)	1.92 (0.70–5.27)	0.203
	GG	6 (27.3)	16 (72.7)	2.05 (0.59–7.15)	0.260
	GT + TT	22 (28.2)	56 (71.8)	1.96 (0.75–5.12)	0.170
*XRCC3* rs1799794	AA	14 (25.9)	40 (74.1)	Ref.	
	AG	11 (28.2)	28 (71.8)	0.89 (0.35–2.25)	0.807
	GG	7 (87.5)	1 (12.5)	0.05 (0.01–0.44)	0.007
	AG + GG	18 (38.3)	29 (61.7)	0.56 (0.24–1.31)	0.185
*XRCC3* rs861539	CC	15 (34.1)	29 (65.9)	Ref.	
	CT	13 (28.9)	32 (71.1)	1.27 (0.52–3.12)	0.598
	TT	4 (33.3)	8 (66.7)	1.03 (0.27–4.00)	0.961
	CT + TT	17 (29.8)	40 (70.2)	1.22 (0.52–2.83)	0.648

CI, confidence interval; HRR, homologous recombination repair; OR, odds ratio; SNP, single nucleotide polymorphism.

**Table 6:** Association of selected polymorphisms in HRR genes with NYHA class.

SNP	Genotype	NYHA 1 N (%)	NYHA 2 N (%)	OR (95% CI)	*p*	OR (95% CI)_adj_	*p*_adj_
*NBN* rs1805794	CC	37 (78.7)	10 (21.3)	Ref.		Ref.	
	CG	41 (89.1)	5 (10.9)	0.45 (0.14–1.44)	0.179	0.31 (0.08–1.25)	0.099
	GG	6 (75.0)	2 (25.0)	1.23 (0.22–7.07)	0.814	0.86 (0.13–5.61)	0.871
	CG + GG	47 (87.0)	7 (13.0)	0.55 (0.19–1.59)	0.269	0.40 (0.12–1.37)	0.145
*NBN* rs709816	AA	30 (76.9)	9 (23.1)	Ref.		Ref.	
	AG	46 (88.5)	6 (11.5)	0.44 (0.14–1.35)	0.149	0.31 (0.08–1.18)	0.086
	GG	8 (80.0)	2 (20.0)	0.83 (0.15–4.65)	0.835	0.54 (0.09–3.44)	0.515
	AG + GG	54 (87.1)	8 (12.9)	0.49 (0.17–1.41)	0.189	0.36 (0.11–1.21)	0.098
*NBN* rs1063054	AA	35 (83.3)	7 (16.7)	Ref.		Ref.	
	AC	40 (85.1)	7 (14.9)	0.88 (0.28–2.74)	0.819	0.91 (0.25–3.25)	0.881
	CC	9 (75.0)	3 (25.0)	1.67 (0.36–7.76)	0.515	1.23 (0.23–6.63)	0.808
	AC + CC	49 (83.1)	10 (16.9)	1.02 (0.35–2.94)	0.970	0.99 (0.30–3.22)	0.982
*RAD51* rs1801320	GG	61 (83.6)	12 (16.4)	Ref.		Ref.	
	GC	23 (82.1)	5 (17.9)	1.11 (0.35–3.48)	0.865	1.01 (0.28–3.62)	0.986
*RAD51* rs1801321	GG	32 (94.1)	2 (5.9)	Ref.		Ref.	
	GT	44 (81.5)	10 (18.5)	3.64 (0.75–17.74)	0.110	4.36 (0.76–25.11)	0.099
	TT	8 (61.5)	5 (38.5)	10.00 (1.63–61.33)	0.013	9.27 (1.28–67.02)	0.027
	GT + TT	52 (77.6)	15 (22.4)	4.62 (0.99–21.52)	0.052	5.41 (0.98–29.80)	0.053
*RAD51* rs12593359	TT	15 (65.2)	8 (34.8)	Ref.		Ref.	
	GT	48 (85.7)	8 (14.3)	0.31 (0.10–0.98)	0.045	0.47 (0.13–1.69)	0.248
	GG	21 (95.5)	1 (4.5)	0.09 (0.01–0.79)	0.030	0.07 (0.01–0.81)	0.034
	GT + TT	69 (88.5)	9 (11.5)	0.25 (0.08–0.74)	0.012	0.31 (0.09–1.05)	0.060
*XRCC3* rs1799794	AA	45 (83.3)	9 (16.7)	Ref.		Ref.	
	AG	32 (82.1)	7 (17.9)	1.09 (0.37–3.24)	0.872	2.07 (0.56–7.59)	0.275
	GG	7 (87.5)	1 (12.5)	0.71 (0.08–6.54)	0.766	1.67 (0.14–19.52)	0.683
	AG + GG	39 (83.0)	8 (17.0)	1.03 (0.36–2.92)	0.962	2.00 (0.57–7.04)	0.279
*XRCC3* rs861539	CC	36 (81.8)	8 (18.2)	Ref.		Ref.	
	CT	37 (82.2)	8 (17.8)	0.97 (0.33–2.87)	0.960	0.89 (0.26–3.09)	0.850
	TT	11 (91.7)	1 (8.3)	0.41 (0.05–3.64)	0.423	0.16 (0.01–2.19)	0.169
	CT + TT	48 (84.2)	9 (15.8)	0.84 (0.296–2.40)	0.750	0.68 (0.20–2.25)	0.523

Adj: adjusted for hyperlipidemia and body mass index. CI, confidence interval; HRR, homologous recombination repair; NYHA, New York Heart Association; OR, odds ratio; SNP, single nucleotide polymorphism.

**Table 7:** Association of selected polymorphisms in HRR genes with late skin adverse events.

		LENT-SOMA					CTCAE v.3				
SNP	Genotype	2/3, N (%)	OR (95% CI)	*p*	OR (95% CI)_adj_	*p*_adj_	2, N (%)	OR (95% CI)	*p*	OR (95% CI)_adj_	*p*_adj_
*NBN* rs1805794	CC	15 (31.9)	Ref.		Ref.		6 (12.8)	Ref.		Ref.	
	CG	14 (30.4)	0.93 (0.39–2.25)	0.878	1.14 (0.43–3.05)	0.788	4 (8.7)	0.65 (0.17–2.48)	0.529	0.75 (0.18–3.13)	0.695
	GG	4 (50.0)	2.13 (0.47–9.71)	0.327	2.93 (0.43–20.07)	0.274	2 (25.0)	2.28 (0.37–13.99)	0.374	3.08 (0.31–30.81)	0.339
	CG + GG	18 (33.3)	1.07 (0.46–2.46)	0.880	1.29 (0.50–3.31)	0.594	6 (11.1)	0.85 (0.26–2.85)	0.798	0.98 (0.26–3.63)	0.974
*NBN* rs709816	AA	12 (30.8)	Ref.		Ref.		6 (15.4)	Ref.		Ref.	
	AG	17 (32.7)	1.09 (0.45–2.67)	0.846	1.22 (0.45–3.33)	0.694	4 (7.7)	0.46 (0.12–1.75)	0.254	0.44 (0.11–1.85)	0.262
	GG	4 (40.0)	1.50 (0.36–6.31)	0.580	2.52 (0.40–16.04)	0.329	2 (20.0)	1.38 (0.23–8.13)	0.725	2.22 (0.22–21.91)	0.496
	AG + GG	21 (33.9)	1.15 (0.49–2.72)	0.746	1.34 (0.51–3.53)	0.555	6 (9.7)	0.59 (0.18–1.98)	0.392	0.59 (0.16–2.198)	0.429
*NBN* rs1063054	AA	12 (28.6)	Ref.		Ref.		3 (7.1)	Ref.		Ref.	
	AC	15 (31.9)	1.17(0.47–2.91)	0.732	1.497 (0.53–4.23)	0.447	6 (12.8)	1.90 (0.45–8.14)	0.386	2.74 (0.54–13.85)	0.222
	CC	6 (50.0)	2.50 (0.67–9.31)	0.172	3.19 (0.74–13.74)	0.119	3 (25.0)	4.33 (0.75–25.11)	0.102	6.69 (0.90–49.51)	0.063
	AC + CC	21 (35.6)	1.38 (0.59–3.25)	0.459	1.79 (0.67–4.76)	0.245	9 (15.3)	2.34 (0.59–9.23)	0.225	3.42 (0.74–15.84)	0.116
*RAD51* rs1801320	GG	24 (32.9)	Ref.		Ref.		10 (13.7)	Ref.		Ref.	
	GC	9 (32.1)	0.97 (0.38–2.46)	0.944	0.98 (0.35–2.78)	0.975	2 (7.1)	0.49 (0.10–2.37)	0.371	0.46 (0.08–2.48)	0.365
*RAD51* rs1801321	GG	9 (26.5)	Ref.		Ref.		2 (5.9)	Ref.		Ref.	
	GT	17 (31.5)	1.28 (0.49–3.31)	0.616	1.46 (0.51–4.18)	0.481	7 (13.0)	2.38 (0.47–12.22)	0.298	2.76 (0.49–15.49)	0.248
	TT	7 (53.8)	3.24 (0.86–12.26)	0.083	2.30 (0.50–10.57)	0.286	3 (23.1)	4.80 (0.70–32.90)	0.110	3.18 (0.38–26.51)	0.285
	GT + TT	24 (35.8)	1.55 (0.62–3.86)	0.345	1.599 (0.58–4.39)	0.362	10 (14.9)	2.81 (0.58–13.61)	0.200	2.86 (0.54–15.12)	0.216
*RAD51* rs12593359	TT	10 (43.5)	Ref.		Ref.		3 (13.0)	Ref.		Ref.	
	GT	17 (30.4)	0.57 (0.21–1.54)	0.267	0.85 (0.27–2.66)	0.785	7 (12.5)	0.95 (0.22–4.06)	0.947	1.43 (0.28–7.23)	0.665
	GG	6 (27.3)	0.49 (0.14–1.70)	0.260	0.52 (0.13–2.096)	0.356	2 (9.1)	0.67 (0.10–4.43)	0.675	0.77 (0.10–5.98)	0.799
	GT + TT	23 (29.5)	0.54 (0.21–1.42)	0.212	0.73 (0.25–2.14)	0.571	9 (11.5)	0.87 (0.22–3.52)	0.845	1.20 (0.26–5.57)	0.821
*XRCC3* rs1799794	AA	12 (22.2)	Ref.		Ref.		5 (9.3)	Ref.		Ref.	
	AG	16 (41.0)	2.44 (0.99–6.02)	0.054	1.82 (0.65–5.095)	0.256	5 (12.8)	1.44 (0.39–5.37)	0.586	0.80 (0.19–3.43)	0.765
	GG	5 (62.5)	5.83 (1.22–28.00)	0.028	10.90 (1.61–73.72)	0.014	2 (25.0)	3.27 (0.52–20.69)	0.209	3.80 (0.44–32.68)	0.224
	AG + GG	21 (44.7)	2.83 (1.19–6.69)	0.018	2.43 (0.92–6.39)	0.073	7 (14.9)	1.72 (0.51–5.82)	0.387	1.07 (0.28–4.11)	0.917
*XRCC3* rs861539	CC	19 (43.2)	Ref.		Ref.		6 (13.6)	Ref.		Ref.	
	CT	13 (28.9)	0.54 (0.22–1.29)	0.162	0.58 (0.21–1.56)	0.278	5 (11.1)	0.79 (0.22–2.81)	0.718	0.83 (0.21–3.33)	0.797
	TT	1 (8.3)	0.12 (0.01–1.01)	0.051	0.11 (0.01–1.10)	0.060	1 (8.3)	0.58 (0.06–5.31)	0.626	0.72 (0.07–7.81)	0.787
	CT + TT	14 (24.6)	0.43 (0.18–1.00)	0.050	0.45 (0.17–1.16)	0.097	6 (10.5)	0.75 (0.22–2.49)	0.633	0.81 (0.22–3.02)	0.755

Adj: adjusted for arterial hypertension and treatment with taxanes. CI, confidence interval; CTCAE v.3., Common Terminology Criteria for Adverse Events, version 3.0; HRR, homologous recombination repair; LENT SOMA, Late Effects in Normal Tissues/Subjective, Objective, Management and Analytic; OR, odds ratio; SNP, single nucleotide polymorphism.

**Table 8:** Association of selected polymorphisms in HRR genes with the occurrence of a new primary tumor.

SNP	Genotype	No New Primary Tumor N (%)	New Primary Tumor N (%)	OR (95% CI)	*p*
*NBN* rs1805794	CC	42 (89.4)	5 (10.6)	Ref.	
	CG	42 (91.3)	4 (8.7)	0.80 (0.20–3.19)	0.752
	GG	8 (100)	0 (0)	/	0.590 *
	CG + GG	50 (92.6)	4 (7.4)	0.67 (0.17–2.66)	0.572
*NBN* rs709816	AA	35 (89.7)	4 (10.3)	Ref.	
	AG	48 (92.3)	4 (7.7)	0.73 (0.17–3.12)	0.670
	GG	9 (90)	1 (10)	0.97 (0.10–9.80)	0.981
	AG + GG	57 (91.9)	5 (8.1)	0.77 (0.19–3.05)	0.707
*NBN* rs1063054	AA	36 (85.7)	6 (14.3)	Ref.	
	AC	44 (93.6)	3 (6.4)	0.41 (0.10–1.75)	0.228
	CC	12 (100)	0 (0)	/	0.319 *
	AC + CC	56 (94.9)	3 (5.1)	0.32 (0.08–1.37)	0.124
*RAD51* rs1801320	GG	65 (89)	8 (11)	Ref.	
	GC	27 (96.4)	1 (3.6)	0.30 (0.04–2.52)	0.268
*RAD51* rs1801321	GG	30 (88.2)	4 (11.8)	Ref.	
	GT	49 (90.7)	5 (9.3)	0.77 (0.19–3.08)	0.706
	TT	13 (100)	0 (0)	/	0.319 *
	GT + TT	62 (92.5)	5 (7.5)	0.60 (0.15–2.42)	0.477
*RAD51* rs12593359	TT	23 (100)	0 (0)	Ref.	
	GT	51 (91.1)	5 (8.9)	/	0.314 *
	GG	18 (81.8)	4 (18.2)	/	0.049 *
	GT + GG	69 (88.5)	9 (11.5)	/	0.114 *
*XRCC3* rs1799794	AA	50 (92.6)	4 (7.4)	Ref.	
	AG	34 (87.2)	5 (12.8)	1.84 (0.46–7.34)	0.389
	GG	8 (100)	0 (0)	/	1.000 *
	AG + GG	42 (89.4)	5 (10.6)	1.49 (0.38–5.90)	0.572
*XRCC3* rs861539	CC	41 (93.2)	3 (6.8)	Ref.	
	CT	41 (91.1)	4 (8.9)	1.33 (0.28–6.33)	0.717
	TT	10 (83.3)	2 (16.7)	2.73 (0.40–18.61)	0.304
	CT + TT	51 (89.5)	6 (10.5)	1.61 (0.38–6.82)	0.520

* calculated using Fisher’s exact test. CI, confidence interval; HRR, homologous recombination repair; OR, odds ratio; SNP, single nucleotide polymorphism.

## Data Availability

All the data are presented within the article and in the Supplementary Materials. Any additional information is available on request from the corresponding author.

## Supplementary Materials

The following supporting information can be downloaded at: https://www.mdpi.com/article/10.3390/cancers14184365/s1, Table S1: Association of selected polymorphisms in HRR genes with NT-proBNP and LVEF reduction; Table S2: Association of *RAD51* haplotypes with NYHA class; Table S3: Association of *XRCC3* haplotypes with LENT-SOMA grade.
