# Supplementary material for: NBN, RAD51 and XRCC3 Polymorphisms as Potential Predictive Biomarkers of Adjuvant Radiotherapy Toxicity in Early HER2-Positive Breast Cancer
Source: Cancers (Basel). 2022 Sep 8;14(18):4365. doi: 10.3390/cancers14184365 (PMC9496855; doi:10.3390/cancers14184365)
Supplement: Supplementary file 1 [file cancers-14-04365-s001.zip › cancers-1832377-supplementary.pdf]

Table S1. Association of selected polymorphisms in HRR genes with NT-proBNP and LVEF reduction.

		NT-proBNP					LVEF reduction		
**SNP**	**Genotype**	**≥125 ng/l, N (%)**	**OR (95 % CI)**	**P**	**OR (95 % CI)$_{adj}$**	**P$_{adj}$**	**Yes; N (%)**	**OR (95 % CI)**	**P**
NBN rs1805794	CC	19 (40.4)	Ref.				6 (12.8)	Ref.	
	CG	12 (26.1)	0.52 (0.22–1.25)	0.145	0.55 (0.23–1.35)	0.195	3 (6.5)	0.48 (0.11–2.03)	0.317
	GG	5 (62.5)	2.46 (0.52–11.52)	0.254	1.82 (0.37–8.99)	0.463	0 (0.00)	/	0.577*
	CG+GG	17 (31.5)	0.68 (0.299–1.53)	0.350	0.68 (0.29–1.57)	0.367	3 (5.6)	0.40 (0.095–1.71)	0.217
NBN rs709816	AA	17 (43.6)	Ref.				5 (12.8)	Ref.	
	AG	14 (26.9)	0.48 (0.198–1.15)	0.099	0.52 (0.21–1.27)	0.152	4 (7.7)	0.57 (0.14–2.27)	0.422
	GG	5 (50.0)	1.29 (0.32–5.21)	0.717	0.98 (0.23–4.16)	0.980	0 (0.0)	/	0.566*
	AG+GG	19 (30.6)	0.57 (0.25–1.31)	0.188	0.58 (0.25–1.37)	0.217	4 (6.5)	0.47 (0.12–1.87)	0.283
NBN rs1063054	AA	16 (38.1)	Ref.				1 (2.4)	Ref.	
	AC	15 (31.9)	0.76 (0.32–1.83)	0.542	0.698 (0.28–1.72)	0.435	7 (14.9)	7.18 (0.84–60.99)	0.071
	CC	5 (41.7)	1.16 (0.32–4.28)	0.823	1.02 (0.27–3.93)	0.975	1 (8.3)	3.73 (0.22–64.47)	0.366
	AC+CC	20 (33.9)	0.83 (0.37–1.899)	0.664	0.76 (0.32–1.78)	0.522	8 (13.6)	6.43 (0.77–53.53)	0.085
RAD51 rs1801320	GG	26 (35.6)	Ref.				6 (8.2)	Ref.	
	GC	10 (35.7)	1.00 (0.40–2.49)	0.993	1.07 (0.42–2.73)	0.880	3 (10.7)	1.34 (0.31–5.77)	0.694
RAD51 rs1801321	GG	13 (38.2)	Ref.				2 (5.9)	Ref.	
	GT	18 (33.3)	0.81 (0.33–1.97)	0.639	0.78 (0.31–1.94)	0.588	6 (11.1)	2.00 (0.38–10.54)	0.414
	TT	5 (38.5)	1.01 (0.27–3.76)	0.989	0.85 (0.22–3.34)	0.812	1 (7.7)	1.33 (0.11–16.09)	0.821
	GT+TT	23 (34.3)	0.84 (0.36–1.99)	0.699	0.79 (0.33–1.91)	0.599	7 (10.4)	1.87 (0.37–9.52)	0.453
RAD51 rs12593359	TT	9 (39.1)	Ref.				2 (8.7)	Ref.	
	GT	17 (30.4)	0.68 (0.25–1.87)	0.452	0.79 (0.28–2.24)	0.653	6 (10.7)	1.26 (0.24–6.76)	0.787
	GG	10 (45.5)	1.296 (0.396–4.24)	0.668	1.44 (0.42–4.92)	0.557	1 (4.5)	0.50 (0.04–5.94)	0.583
	GT+TT	27 (34.6)	0.82 (0.32–2.15)	0.691	0.95 (0.35–2.55)	0.912	7 (9.0)	1.04 (0.20–5.36)	0.967
XRCC3 rs1799794	AA	19 (35.2)	Ref.				4 (7.4)	Ref.	
	AG	14 (35.9)	1.03 (0.44–2.44)	0.944	1.11 (0.46–2.68)	0.824	5 (12.8)	1.84 (0.46–7.34)	0.389
	GG	3 (37.5)	1.11 (0.24–5.14)	0.898	1.28 (0.26–6.35)	0.765	0 (0.0)	/	1.000*
	AG+GG	17 (36.2)	1.04 (0.46–2.36)	0.918	1.13 (0.49–2.63)	0.773	5 (10.6)	1.49 (0.38–5.899)	0.572
XRCC3 rs861539	CC	17 (38.6)	Ref.				4 (9.1)	Ref.	
	CT	14 (31.1)	0.72 (0.299–1.72)	0.457	0.73 (0.29–1.79)	0.485	3 (6.7)	0.71 (0.15–3.39)	0.672
	TT	5 (41.7)	1.13 (0.31–4.16)	0.849	1.30 (0.34–4.93)	0.697	2 (16.7)	2.00 (0.32–12.51)	0.459
	CT+TT	19 (33.3)	0.79 (0.35–1.80)	0.581	0.83 (0.36–1.92)	0.656	5 (8.8)	0.96 (0.24–3.81)	0.956

Adj: adjusted for age; *calculated using Fisher's exact test

CI, confidence interval; HRR, homologous recombination repair; LVEF, left ventricular ejection fraction NT-proBNP, N-terminal pro B-type natriuretic peptide; OR, odds ratio; SNP, single nucleotide polymorphism

Table S2. Association of *RAD51* haplotypes with NYHA class.

Haplotype[1]	Estimated frequency		OR (95 % CI)	P	OR (95 % CI)$_{adj1}$	P$_{adj1}$	OR (95 % CI)$_{adj2}$	P$_{adj2}$
	NYHA 1	NYHA 2						
GGG	0.497	0.235	Ref.					
GTT	0.325	0.529	4.27(1.45–12.58)	0.009	3.69 (1.24–11.02)	0.019	4.37 (1.33–14.35)	0.015
CGT	0.110	0.147	2.39(0.66–8.65)	0.183	2.30(0.59–8.95)	0.231	2.01(0.47–8.65)	0.350

[1]SNP order from 5′- to 3′-end: rs1801320, rs1801321, rs12593359

Adj1: adjusted for body mass index; Adj2: adjusted for hyperlipidemia

CI, confidence interval; NYHA, New York Heart Association; OR, odds ratio; SNP, single nucleotide polymorphism

Table S3. Association of *XRCC3* haplotypes with LENT-SOMA grade.

Haplotype[1]	Estimated frequency		OR (95 % CI)	P	OR (95 % CI)$_{adj}$	P$_{adj}$
	LENT-SOMA 1	LENT-SOMA 2/3				
AC	0.390	0.379	Ref.			
AT	0.397	0.227	0.57 (0.26-1.27)	0.167	0.57 (0.23-1.40)	0.219
GC	0.213	0.394	1.93 (0.94-3.98)	0.074	2.06 (0.87-4.85)	0.100

[1]SNP order from 5′- to 3′-end: rs1799794, rs861539

Adj: adjusted for arterial hypertension and treatment with taxanes

CI, confidence interval; LENT SOMA, Late Effects in Normal Tissues/Subjective, Objective, Management and Analytic; OR, odds ratio; SNP, single nucleotide polymorphism
